# Very rare case of rectal carcinosarcoma

**DOI:** 10.1007/s10151-012-0947-x

**Published:** 2012-11-22

**Authors:** M. Kołodziejczak, K. Bielecki, I. Sudoł-Szopińska, A. Szcześniak, A. Obcowska

**Affiliations:** 1 Department of General Surgery, Proctology Unit, Solec Hospital, 93 Solec str., 00-382 Warsaw, Poland; 2 Department of Diagnostic Imaging, Institute of Rheumatology, Warsaw, Poland; 3 Warsaw and Department of Diagnostic Imaging, Warsaw Medical University, Warsaw, Poland; 4 Pathomorphology Department, Solec Hospital, Warsaw, Poland; 5 Department of General Surgery, Praski Hospital, Warsaw, Poland

Dear Sir,

Carcinosarcoma is a rare type of malignant neoplasm, which was first described by Virchow in 1864. It is composed of two types of neoplastic cells, epithelial, and mesenchymal ones. This neoplasm has a poor prognosis and may develop in many organs, including the uterus and ovaries of older women, the head and neck region, and the lungs and kidneys. Within the gastrointestinal tract, carcinosarcoma is most commonly found in the esophagus [1] and only rarely in the large intestine. Among case reports before 2011, 16 fulfilled the criteria for carcinosarcoma of the large intestine, and of these, only 5 involved the rectum. We report a case of carcinosarcoma of the rectum with necrosis of the intestinal wall.

**Fig. 1:**
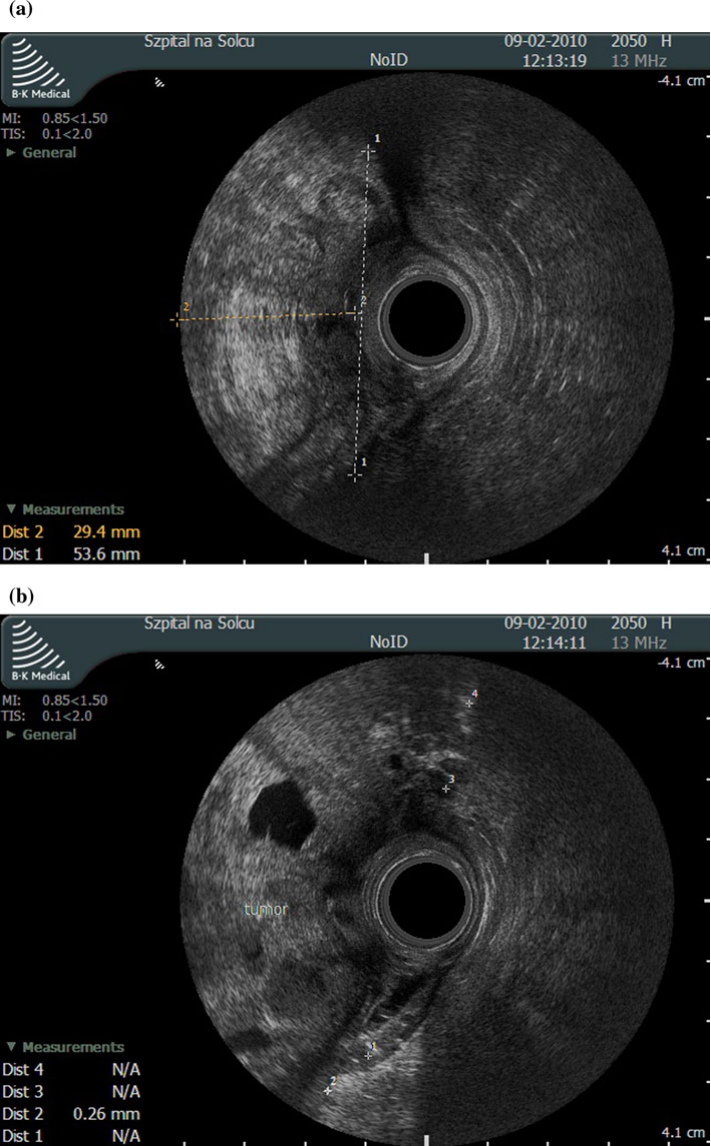
**a** The endosonographic study shows a hyperechoic tumor, measuring 30 × 53 mm in a transversal plane, taking up the* right half* of the anal canal lumen, infiltrating the anal sphincters, **b** With anechoic areas of necrosis

An 83-year-old male was admitted to the surgical ward with signs of massive lower gastrointestinal tract bleeding. The patient had congestive heart failure, diabetes, and chronic respiratory failure secondary to venous thromboembolism. On digital rectal examination, a stenotic, bleeding tumor of the rectum, infiltrating the perianal tissues on the right side could be felt. The distal margin of the tumor was palpable at the level of anorectum, while its superior margin was unreachable. Anal endosonography revealed a tumor (30 mm × 53 mm × 40 mm in size) occupying the right half of the anal canal, infiltrating the sphincter muscles (internal and external), with areas of necrosis (Fig. 1).

Due to the deterioration of the patient’s overall condition and continued signs of active bleeding from the tumor, the patient qualified for emergency surgery (ASA IV).

Intraoperatively, on rectal examination, a stenotic tumor with an ulceration at the right side of the rectal wall was confirmed. The necrotic tissue was partially removed, the rectum was rinsed with normal saline, then, the site of bleeding was coagulated, and internal packing of the rectum was carried out. An exploratory laparotomy was performed through an infraumbilical midline incision. A loop sigmoidostomy was created due to necrosis of the rectal wall and perirectal tissue. The remaining abdominal organs did not reveal any abnormalities on palpation (e.g., distant metastases). There were no complications in the early postoperative period. However, due to the patient’s poor overall condition, he was not a candidate for oncological treatment. The patient was discharged home on postoperative day 14 and advised to return for the histopathology results. He died 5 weeks after the surgery, due to cardiorespiratory failure.

**Fig. 2:**
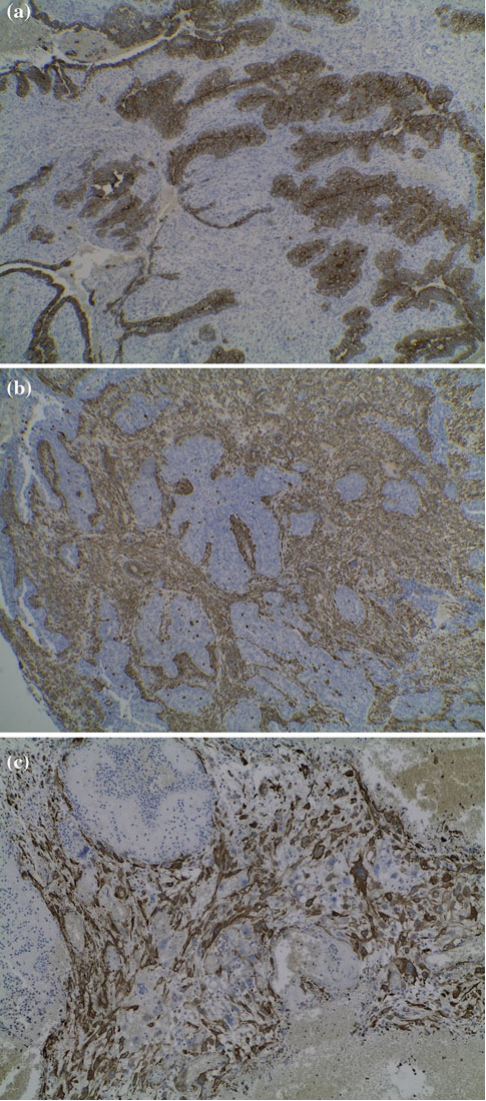
**a** Staining using anti-cytokeratin antibodies used to identify cells of epithelial origin; **b** Staining using anti-vimentin antibodies to identify cells of mesenchymal-origin; **c** Staining using anti-SMA (smooth muscle antigen) antibodies

The histopathology examination of the specimen revealed carcinosarcoma of the rectum which was confirmed by immunohistochemical staining which was positive for cytokeratin, vimentin, and smooth muscle antigen (Fig. 2). This mixed neoplasm was formed from epithelial elements of a well-differentiated adenocarcinoma as well as from mesenchymal neoplastic elements, showing differentiation toward muscle tissue.

Carcinosarcoma of the colon is a rare neoplasm composed of both carcinomatous and sarcomatous elements. It has also been called as sarcomatoid carcinoma, carcinoma with mesenchymal stroma, carcinoma with sarcomatous change, spindle cell carcinoma, and pleomorphic anaplastic carcinoma [2]. The prognosis of this carcinoma is poor because it invades deep into the bowel wall, has widespread metastases, and is resistant to multi-agent chemotherapy. Up to now, all the described cases of rectal carcinosarcoma included lymph node metastases at the time of diagnosis [3–5]. Our patient did not have any distant metastases noted at explorative laparotomy. However, the local spread of the neoplasm led to necrosis of a fragment of the rectal wall and secondary bleeding, a complication which had not been described previously. The only published case with necrosis of the intestinal wall involved the carcinosarcoma of the ascending colon [6].

Survival in the reported cases of rectal carcinosarcoma was never more than 6 months. Our patient died within a mere 5 weeks, which should be attributed to the advanced local state of the cancer at the time of diagnosis and the patient’s poor general condition which prevented further treatment. The case presented in this article is, to the best of our knowledge, the first involving bleeding into the gastrointestinal tract secondary to perforation of the rectal wall as a complication associated with this neoplasm. Therefore, the rare diagnosis of carcinosarcoma should be considered in patients with gastrointestinal bleeding.
